# Mechanical Venous Thrombectomy Using Indigo Aspiration System: A Case Report

**DOI:** 10.7759/cureus.38241

**Published:** 2023-04-28

**Authors:** Riichi A Ota, Gabriel Neves, Victor C Montalvan, Thomas Windisch, Saif Bushnaq

**Affiliations:** 1 Neurology, Texas Tech University Health Sciences Center, Lubbock, USA; 2 Radiology, Covenant Health, Lubbock, USA

**Keywords:** vascualr neurology, stroke, endovascular thrombectomy, penumbra’s indigo aspiration system, cerebral venous sinus thrombosis (cvst)

## Abstract

We present a case of successful endovascular thrombectomy of cerebral venous sinus thrombosis utilizing Penumbra’s Indigo Aspiration System (Penumbra Inc., Place Alameda, CA), a minimally invasive system with a large-lumen (Indigo System CAT7, 7F) catheter predominantly used for the removal of thromboembolism involving the peripheral arterial and venous systems. A 30-year-old female presented with a seizure and focal neurological deficits and was found to have a left posterior temporal lobe hemorrhagic infarct secondary to an extensive cerebral venous sinus thrombosis extending from the left transverse sinus to the ipsilateral internal jugular bulb. We considered the combination of seizure, motor deficit, and hemorrhagic infarct high-risk features for poor response to standard medical therapy with therapeutic anticoagulation. Therefore, we performed a mechanical venous thrombectomy with the above device in addition to anticoagulation treatment with heparin infusion. This combination therapy resulted in a technically successful radiographic recanalization of the involved sinuses and an excellent functional outcome at follow-up. This case demonstrates that this trackable, atraumatic, large-bore system was safe and efficacious in the cerebral venous system, permitting near-complete thrombus removal.

## Introduction

Commonly utilized aspiration catheters in neuro-interventional procedures for cerebral venous sinus thrombosis (CVST) are the AngioJet device, clot retraction with Penumbra System, balloon venoplasty with or without stenting, and others [1-3]. Penumbra’s Indigo Aspiration System (Penumbra Inc., Place Alameda, CA) is a trackable, large-lumen (Indigo System CAT7, 7F) catheter and aspiration system used for aspiration of thromboembolism involving the peripheral venous and arterial systems, as well as pulmonary emboli. A single case report in the literature used the same system for CVST, limited by its short case description and follow-up [4]. A young female with CVST presented with seizure, quadrantanopia, aphasia, and intracranial venous hemorrhagic infarct. According to a recent study, we considered the presence of seizure, focal neurological deficit, and hemorrhage as a high-risk feature for poor response to standard medical therapy with anticoagulation [5]. Therefore, we proceeded to endovascular venous thrombectomy (EVT). Here, we show that this large-lumen system may safely retrieve and successfully recanalize CVST, addressing the problem of available tools for complete thrombus removal.

## Case presentation

A 30-year-old right-handed female presented with one week of left ear pain, recurrent transient episodes of trouble selecting words to describe situations, problems visualizing objects on her right, and left occipital headache. Her husband also reported an episode of a generalized tonic-clonic seizure lasting for three minutes during sleep which was associated with tongue laceration the night before admission. She had a past medical history of migraine without aura, and her family history revealed that her maternal grandmother had recurrent ischemic strokes. She denied any personal or family history of hypercoagulability disorder, tobacco abuse, oral contraceptive use, current pregnancy, or recent head trauma. Her initial physical examination revealed left superior quadrantanopia and anomia but no focal motor or sensory deficit in any extremities. A direct fundoscopy did not reveal papilledema.

**Figure 1:**
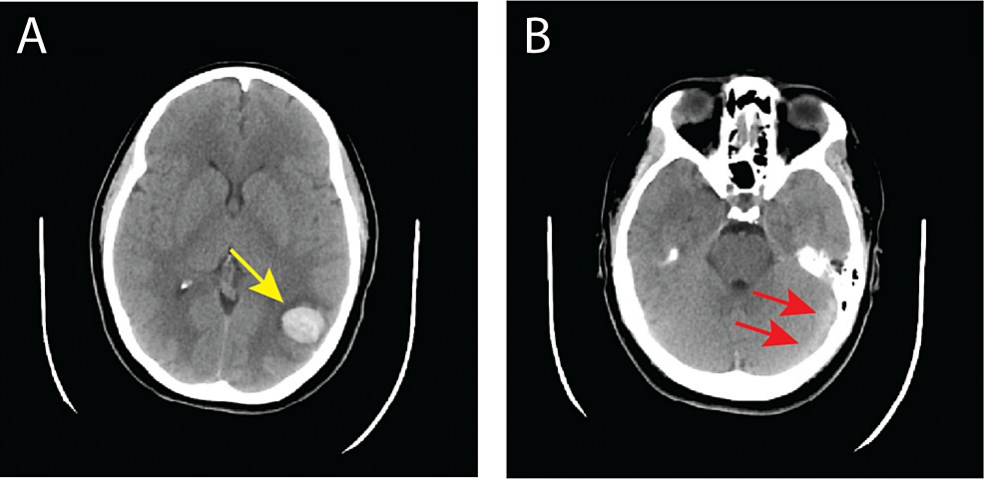
Initial non-contrast CT scan of the head demonstrating an intraparenchymal hemorrhage (A; yellow arrow) due to cerebral venous sinus thrombosis evidenced by the hyperdense left transverse sinus (B; red arrows).

**Figure 2:**
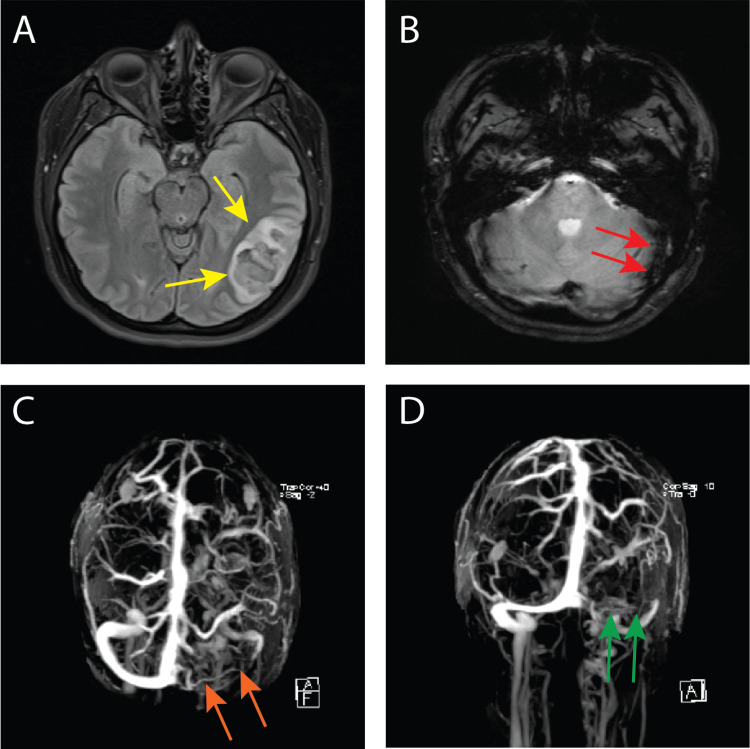
Findings on magnetic resonance imaging and magnetic resonance venography of the brain. Magnetic resonance imaging confirms the diagnosis of intraparenchymal hematoma (IPH) secondary to cerebral venous sinus thrombosis. (A) A T2 fluid-attenuated inversion recovery image is showing the left posterior temporal IPH associated with perihematomal edema (yellow arrows). (B) A gradient-related echo image is showing hypointensity involving the left transverse and sigmoid sinus (red arrows). Axial (C; orange arrows) and coronal (D; green arrows) reconstructions of magnetic resonance venography with contrast showing paucity of contrast in thrombosed sinuses.

An unenhanced head CT scan revealed an intraparenchymal hemorrhage in the left posterior temporal lobe (intracerebral hemorrhage score 0) associated with a hyperdense left transverse sinus (Figures 1A, 1B). A magnetic resonance imaging and magnetic resonance venogram of the brain with and without contrast revealed local perihematomal edema and increased susceptibilities involving the torcula, left transverse sinus, sigmoid sinus, and jugular bulb consistent with a subacute CVST (Figures 2A-2D). A routine scalp electroencephalogram showed intermittent focal slowing of the left temporal region but did not reveal evidence of epileptogenicity.

**Figure 3:**
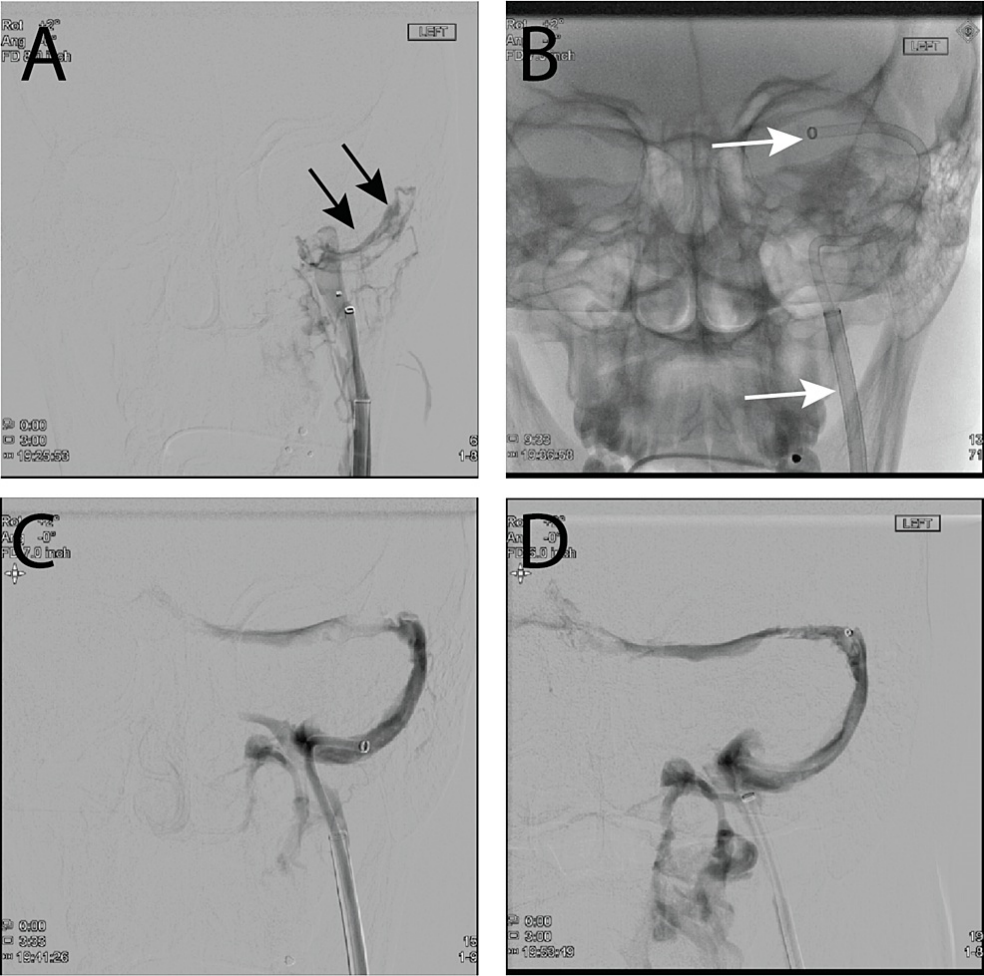
Digitally subtracted angiography of the cerebral venous system. Cerebral digitally subtracted angiography (DSA) acquired during the procedure is demonstrating results using Penumbra’s Indigo Aspiration System. (A) A cerebral DSA is showing in situ thrombus in the left sigmoid sinus (black arrows). (B) An unsubtracted cerebral DSA showing the Indigo 7 French aspiration catheter in the left transverse sinus (top white arrow) and proximal 12 French aspiration catheter in the ipsilateral internal jugular vein (bottom white arrow). Post-aspiration DSA following 1 (C) and 4 (D) aspiration attempts.

Given the patient’s clinical presentation with seizure, focal neurological deficits, and intracranial hemorrhage, we considered the patient at high risk for poor response to standard medical therapy with anticoagulation [5]. Therefore, after a thorough discussion with the patient about the risk and benefit profile of the procedure, we proceeded to EVT after we obtained verbal and written consent. We obtained venous access using 12F Gore DrySeal Flex Sheath (W. L. Gore & Associates, Newark, DE). Subsequently, we advanced the sheath over Bernstein Penumbra Select Catheter (Penumbra Inc., Place Alameda, CA) under fluoroscopic guidance, and we parked the sheath at the skull base. The left transverse sigmoid sinuses were accessed using a Fathom guidewire (Boston Scientific, Marlborough, MA) and Phenom 27 microcatheter (Medtronic, Minneapolis, MN). After advancing the aspiration catheter, we performed an aspiration thrombectomy using a 7 French (F) Indigo System guide catheter (CAT7) within a 12 F large lumen catheter. Aspiration was applied using the Lightning Intelligent Aspiration System (Penumbra Inc., Place Alameda, CA). Figures 3A-3D illustrate the near-total thrombus removal following aspiration attempts permitting immediate flow restoration in the previously occluded venous sinus. The patient was admitted to the neurological ICU for neuromonitoring, and we continued a continuous therapeutic heparin infusion (goal PTT 70-90) for two days before transitioning to oral apixaban. We also started the patient on levetiracetam for seizure treatment. The patient remained seizure-free in the hospital and endorsed notable improvement of the headache the day following the procedure and improvement of the vision. Her anomia was persistent at discharge. She was discharged home in stable condition with the continuity of the same regimen of levetiracetam and apixaban and instructed to present for a clinical follow-up four weeks from discharge.

At clinical follow-up, the patient expressed complete resolution of headache, anomia, and blurry vision. She also remained seizure-free.

## Discussion

The thrombolysis or anticoagulation for cerebral venous thrombosis (TO-ACT) trial is the only randomized study that evaluated the efficacy and safety of EVT in CVST; however, in the study, EVT did not lead to significant improvement of functional outcome [3]. Despite the lack of robust evidence, European Stroke Association and American Heart Association guidelines recommend considering EVT on patients with CVST with a high probability of poor clinical outcomes and those who continue to deteriorate despite standard medical therapy [6,7]. A recent single-center retrospective cohort study revealed that patients with CVST with extreme ages, clinical deterioration after admission, low Glasgow Coma Scale, focal motor deficit, seizure, intracerebral hemorrhage, involvement of superior sagittal sinus, or bilateral transverse sinuses had poor response to anticoagulation [5]. In our case, the patient had a pre-treatment high risk of poor outcomes, including seizure, intracerebral hemorrhage, and extensive sinus involvement. Therefore, we decided to proceed with EVT.

Conventionally, the techniques and devices used in CVST are the AngioJet device, balloon venoplasty with or without stenting, Penumbra ACE system, stent retriever, and others [1-3]. Table 1 summarizes recent case reports with devices used for venous thrombectomy. The diameter of the aspiration catheter for thrombectomy does not traditionally exceed 6 F for the cerebral sinus system or 8 F for the internal jugular vein [1,2,8]. We utilized 7 F Penumbra’s Indigo Aspiration System, a large-lumen device that more closely matches the diameter of the most prominent cerebral sinuses [1]. The additional benefit of this device is its Lightening Intelligent Aspiration Tubing. It has a dual pressure sensor that, when the clot is engaged, opens the tubing valve, and enlightens the system providing visual cues. On the contrary, when the clot is not engaged, the valve is closed, minimizing blood loss, and optimizing the procedure.

**Table 1:** Summary of recent case reports with devices used for venous thrombectomy

First author, year of publication	Age (years), sex	Clinical presentation	Indication for thrombectomy	Sinuses involved	Devices used	Diameter of catheters (if applicable)
Zhou et al, 2022 [9]	29, female	8-week pregnancy, headache, nausea, vomiting	Development of focal motor deficit despite anticoagulation	Superior sagittal sinus, transverse sinus, sigmoid sinus	Stent retriever (Solitaire, Medtronic)	N/A
Nakagawa et al, 2022 [10]	43, male	Severe headache, seizure, intraparenchymal hemorrhage	Development of focal motor deficit despite anticoagulation	Superior sagittal sinus, transverse sinus	Balloon angioplasty (Rapid cross, Medtronic), stent retriever (Trevo, Stryker), and aspiration catheter (ACE 68, Penumbra)	0.068 inch (ACE 68)
Crouch et al, 2022 [11]	69, male	Seizure, altered mental status	Development of hydrocephalus	Superior sagittal sinus, torcula, bilateral transverse sinus, bilateral sigmoid sinus, and straight sinus	Stent retriever (Trevo, Stryker), and aspiration catheter (name not mentioned)	N/A
Lau et al, 2020 [12]	32, female	Headache, altered mental status	Lack of clinical improvement despite anticoagulation	Superior sagittal sinus, straight and left transverse sinus	Stent retriever (Solitaire, Medtronic), aspiration catheter (ACE 64, Penumbra)	0.064 inch (ACE 64)
Liao et al, 2015 [8]	29, male	Headache, vomiting, and focal motor deficit	Development of seizure, the decline in mental status, and development of intracranial hypertension	Superior sagittal sinus, and transverse sinus	Aspiration catheter (0.054-inch Penumbra System, Penumbra)	0.054 inch
Liao et al, 2015 [8]	27, female	Headache, vomiting, focal motor deficit after abortion	Development of intracranial hypertension despite anticoagulation	Superior sagittal sinus	Aspiration catheter (0.041-inch Penumbra System, Penumbra)	0.041 inch

While most endovascular devices used in interventions for cerebral venous vasculature are designed for arterial vasculature, the application of systems designed for peripheral venous vasculature can be safely applied in the cerebral venous sinuses, as described here. In this case, we describe a new application for Penumbra’s Indigo System by performing a successful venous thrombectomy using a large-lumen 7 F catheter to perform EVT for CVST, resulting in a near-total recanalization of the thrombosed sinuses with an initial pass and good functional outcome on follow-up.

## Conclusions

Venous sinus recanalization is an important outcome measure in trials investigating endovascular therapies for CVST. Most endovascular tools used for aspiration of CVST are designed for arterial, not venous, vasculature. Penumbra’s Indigo Aspiration System, a large-lumen catheter, can be considered a safe and effective alternative for treating CVST. Future efforts should continue to investigate practical tools for endovascular intervention in CVST, such as the Indigo Aspiration System, to improve patient outcomes and sufficiently answer the question about the efficacy and safety of endovascular therapies for CVST.
